# Enzyme-Catalyzed Synthesis of Water-Soluble Conjugated Poly[2-(3-thienyl)-Ethoxy-4-Butylsulfonate]

**DOI:** 10.3390/polym8040139

**Published:** 2016-04-13

**Authors:** Yun Zhao, Hongyan Zhu, Xinyang Wang, Yingying Liu, Xiang Wu, Heyuan Zhou, Zhonghai Ni

**Affiliations:** School of Chemical Engineering and Technology, China University of Mining and Technology, Xuzhou 221116, China; zhuhongyan2014@126.com (H.Z.); 18796287559@163.com (X.W.); 18361226016@163.com (Y.L.); fangsiaixiedi@163.com (X.W.); zhouheyuanwork@163.com (H.Z.)

**Keywords:** water-soluble polythiophene, enzyme-catalyzed polymerization, hybrid solar cell

## Abstract

An environmentally friendly water-soluble conjugated polythiophene poly[2-(3-thienyl)-ethoxy-4-butylsulfonate] (PTEBS) has been found to be effective for making hybrid solar cells. In this work, we first report the enzyme-catalyzed polymerization of (3-thienyl)-ethoxy-4-butylsulfonate (TEBS) using horseradish peroxidase (HRP) enzyme as a catalyst and hydrogen peroxide (H_2_O_2_) as an oxidant in an aqueous buffer. This enzyme-catalyzed polymerization is a “green synthesis process” for the synthesis of water-soluble conjugated PTEBS, the benefits of which include a simple setting, high yields, and an environmentally friendly route. Fourier transform infrared spectra (FTIR) and UV–Vis absorption spectra confirm the successful enzyme-catalyzed polymerization of TEBS. The thermo gravimetric (TG) data show the obtained PTEBS is stable over a fairly high range of temperatures. The present PTEBS has a good solubility in water and ethanol, and photoluminescence quenching of PTEBS/titanium dioxide (TiO_2_) composite implies that the excitons dissociate and separate successfully at the interface of PTEBS and TiO_2_, which help to build solar cells using green processing methods.

## 1. Introduction

Conjugated polythiophenes have received significant attention recently due to their nonlinear optical properties, electro-conductivity, and other valuable properties. They can be employed in electrical components such as organic field-effect transistors (OFETs) and organic solar cells (OSCs) [1,2,3,4,5]. However, the processability and solubility of unsubstituted polythiophene is poor. The solubility in organic media can be markedly enhanced by introducing flexible alkyl chains, alkoxy groups, or other groups into the polymer backbone, which allow the wet solution preparation of thin film electrical devices via different coating and printing techniques [6,7].

Alternatively, the hydrophilic side chains consist of charged groups, such as phosphonates, sulfonates, or carboxylates groups, that have been attached to polythiophenes to render the polymer water-soluble [8,9,10]. The function of using water as the solvent for the device fabrication process offers several advantages, such as environmentally friendly processing, which avoids toxic organic solvents, careful control of the evaporation of water using heat, which benefits the film morphology and improves stability of the devices under atmospheric conditions. Several works have already been reported OSCs fabricated from water-soluble poly[2-(3-thienyl)-ethoxy-4-butylsulfonate] (PTEBS) [11,12]. Traditionally, PTEBS has been synthesized by chemical oxidation methods [13,14].

Recently, enzymatic polymerization has been explored as an alternative approach to the synthesis of polymers [15,16,17,18,19]. The enzymes offer several advantages such as high selectivity, mild operating conditions, catalyst recyclability, and biocompatibility, which render them environmentally friendly alternatives over conventional chemical catalysts. These characteristics are indicative of the “green synthesis process” nature of the enzymatic catalysis for developing new polymeric materials. Several oxidoreductases (e.g., peroxidase, laccase, bilirubin oxidase, *etc.*) have been reported to catalyze the oxidative polymerization of –OH and –NH_2_ functionalized aromatic compounds [20,21,22]. Among them, horseradish peroxidase (HRP) is the most widely used biocatalyst for the polymerization of polyaromatic compounds such as phenols and anilines in the presence of hydrogen peroxide (H_2_O_2_) as the oxidant.

In this work, we first report the enzyme-catalyzed synthesis of water-soluble conjugated polythiophene PTEBS using HRP as a catalyst and H_2_O_2_ as an oxidant. This enzyme-catalyzed polymerization is a “green synthesis process” for the synthesis of water-soluble conjugated PTEBS, the benefits of which include a simple setting, high yields, and an environmentally friendly route.

## 2. Materials and Methods

### 2.1. General Considerations and Materials

Horseradish peroxidase (HRP, EC1.11.1.7, ≥250 units/mg, solid) were obtained from Sigma-Aldrich Co. (St. Louis., MO, USA) and were used without further purification. All the other chemicals were purchased from Sinopharm Chemical Reagent Co., Ltd. (Shanghai, China) and were of reagent grade. Dichloromethane was distilled from calcium hydride. The 4 Å molecular sieves were activated and stored in an oven at 200 °C until use.

### 2.2. Analytical Measurements

^1^H NMR spectra were collected on a Bruker-400 MHz spectrometer in D_2_O solutions with TMS as an internal standard (Bruker Corporation, Fällanden, Switzerland). The Fourier transform infrared (FTIR) measurements were recorded from KBr pellets by use of a Thermo Nicolet 750 FTIR spectrometer (Artisan Technology Group, Champaign, IL, USA). The weight-average molecular weight was estimated by P230 Gel Permeation Chromatography (GPC) (Elite, Dalian, China, column: SEC-150, XIYU Tech, Shanghai, China) with standard polystyrene as a reference using water as an eluent at 40 °C. UV–Vis absorption spectra were recorded on a Shimadzu UV-3600 UV–Vis–NIR spectrometer (Shimadzu Scientific Instruments, Kyoto, Japan). Emission spectra were performed by a Hitachi F-4600 fluorescence spectrometer (Hitachi High-Technologies Corporation, Tokyo, Japan). Thermo gravimetric (TG) analyses were performed on a TGA/SDTQ600 instrument (TA Instruments-A Division of Waters Ltd., Zellik, Belgium) at a heating rate of 20 °C/min under nitrogen.

### 2.3. Synthesis of Monomer (3-thienyl)-Ethoxy-4-Butylsulfonate (TEBS)

**Figure 1:**
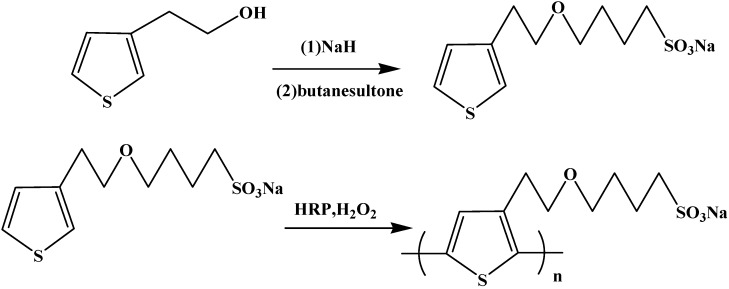
Scheme of enzyme-catalyzed polymerization of water-soluble (3-thienyl)-ethoxy-4-butylsulfonate (TEBS) with horseradish peroxidase (HRP).

The monomer (3-thienyl)-ethoxy-4-butylsulfonate (TEBS) was synthesized by the method proposed in the literature [14]. To a mixed solution of 3-thienyl ethanol (5 mL; 27 mmol) and freshly distilled toluene (200 mL), 1.31 g of sodium hydride (55 mmol) was added slowly, which led to a white precipitate. The resultant mixture was stirred at room temperature for 30 min, followed by 6.17 g of butanesultone (45 mmol) was added drop-wise, and the mixture was then refluxed for 2 h under the protection of nitrogen. The final pale yellow precipitate was obtained by filtration, washed with toluene, and dried under vacuum at 40 °C in 86% yield. The procedure is schematically depicted in Figure 1. UV–Vis: H_2_O, max: 230 nm. IR: KBr, (cm^−1^): 3079 (w, =C–H), 2927 (as, C–H), 2865 (s, C–H), 1453 (sh, CH_2_), 1206 (s, S=O), 1065 (s, C–O–C). ^1^H NMR (D_2_O) (TMS, ppm): 7.44 (m, 1H), 7.20 (m, 1H), 7.11 (m, 1H), 3.78 (t, 2H), 3.57 (t, 2H), 2.94 (t, 2H), 2.91 (t, 2H), 1.69–1.76 (m, 4H) (Figure S1).

### 2.4. Enzyme-Catalyzed Polymerization of TEBS

The polymerization of TEBS was catalyzed by HRP with a hydrogen peroxide (H_2_O_2_) oxidant, which is schematically depicted in Figure 1. Firstly, 2.8 g of monomer TEBS (11 mmol) was dissolved in 4 mL of distilled water at room temperature. Next, the pH was adjusted to 3.0 with the addition of hydrochloric acid, and the temperature was adjusted to 5 °C. A stock solution of HRP (3 mg/mL) was also prepared in distilled water, and 0.15 mL of a HRP solution were added to the typical reaction mixture under magnetic stirring. The polymerization was initiated by the addition of an H_2_O_2_ solution accompanied with the change of color of the reaction solution from faint yellow to red. The H_2_O_2_ solution (1 mmol, 30 wt %) was added to the mixture every 10 min 11 times to prevent inhibition of the enzyme activity. The reaction was carried out at 5 °C for 16 h, and the solution was then transferred to degradable natural cellulose membrane bags (molecular weight cut off 4000 Da) and was dialyzed against 1000 mL of distilled water. Dialysis was carried out for 48 h with distilled water, being changed every 6 h to expedite the removal of enzyme, oligomers, and unreacted monomer. The red brown polymer was achieved by removing the solvent by distillation in vacuum at 40 °C for 24 h. UV–Vis: H_2_O, max: 287 and 351 nm. IR: KBr, (cm^−1^): 2925 (as, C–H), 2857 (s, C–H), 1460 (sh, CH_2_), 1206 (s, S=O), 1065 (s, C–O–C). ^1^H NMR (D_2_O) (TMS, ppm): 3.51 (t, 2H), 3.44 (t, 2H), 2.89 (t, 2H), 2.82 (t, 2H), 1.54–1.70 (m, 4H) (Figure S2).

## 3. Results and Discussion

### 3.1. The Successful Enzyme-Catalyzed Polymerization of TEBS

Following a synthetic procedure described in a recent publication [14], the monomer (3-thienyl)-ethoxy-4-butylsulfonate (TEBS) was easily synthesized in one step with a good yield (86%) from the commercially available 3-thienyl ethanol. Then the oxidative polymerization was carried out in water using horseradish peroxidase (HRP) enzyme as a catalyst, and hydrogen peroxide (H_2_O_2_) as an oxidant. The gravimetric yield of polymerization was 53% (Table 1, entry 1). It was reported that acidic media is necessary for enzyme-catalyzed polymerization [16,23]. The protonic acids used here catalyzed the equilibrium reaction to the corresponding polymer and increased the yield of the polymerization. However, it is known that the activity of the HRP enzyme would decrease in acidic media. Thus, the temperature should be kept lower (5 °C) to decrease the degradation of the HRP enzyme. We had carried out the enzymatic polymerization at room temperature following the same procedure; however, the yield decreased to 40% (Table 1, entry 2).

Different reaction conditions were also studied to establish the optimum one for the enzyme-catalyzed synthesis of poly[2-(3-thienyl)-ethoxy-4-butylsulfonate] (PTEBS), as shown in Table 1. As we can see, the enzymatic polymerization, which was carried out following the same procedure but with five times the amount of HRP, the yield changed little (entry 1–3). However, it would have caused too much time to dialyze for the removal of enzyme. The reaction time was also adjusted from 16 to 24 h to increase the yield; however, the yield did not obviously increase (entry 1–4). As a result, we terminated the reaction at 16 h for convenience. The amount of H_2_O_2_ was also investigated. Experiments were run with three different molar ratios of TEBS and H_2_O_2_ (2:1, 1:1, 1:2; entry 5, 1, 6, respectively). An increased yield from 21% to 53% was observed, when the ratio of TEBS and H_2_O_2_ changed from 2:1 to 1:1. When the ratio of oxidant was two times the monomer, the yield started to decrease. We demonstrated that one molecule of the monomer TEBS requires one molecule of the oxidant H_2_O_2_ for polymerization. The higher concentration of H_2_O_2_ likely inhibited the HRP enzyme activity.

**Figure 2:**
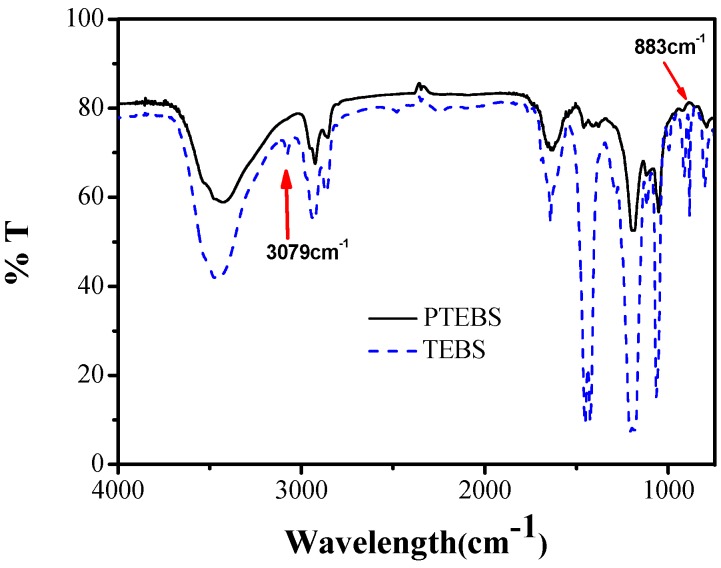
Fourier transform infrared (FTIR) spectra of monomer TEBS and polymer poly[2-(3-thienyl)-ethoxy-4-butylsulfonate] (PTEBS).

However, the molecular weight of the obtained PTEBS is low. Gel permeation chromatography (GPC) measurements revealed a weight-average molecular weight of 1650 g/moL with a polydispersity index of 1.2 (Figure S3). In recent years, we have shown that the synthesis of conducting polymers such as poly(3,4-ethylenedioxythiophene) (PEDOT) can be carried out enzymatically in a good yield [23]. However, TEBS is not as easily polymerized as 3,4-ethylenedioxythiophene (EDOT) without its electron-donating oxygen atoms, which can decrease the oxidation potential. Therefore, the molecular weight remains limited. The chemical natural of the polymer was studied by FTIR spectroscopy. Figure 2 shows the FTIR spectra of monomer TEBS and as-synthesized polymer PTEBS. A peak at 3079 cm^−1^ in the FTIR spectra of monomer TEBS is due to the C–H bond stretching in the thiophene ring. The most significant feature, however, was the absence of a peak at 3079 cm^−1^ for the spectrum of PTEBS, proving the successful enzyme-catalyzed polymerization of PTEBS. Furthermore, the absorption peak at 883 cm^−1^ ascribable to the C–Hα out-of-plane deformation of thiophene rings is found to be rather weak, which indicating that the thiophene rings were polymerized by α–α′ coupling [24].

**Figure 3:**
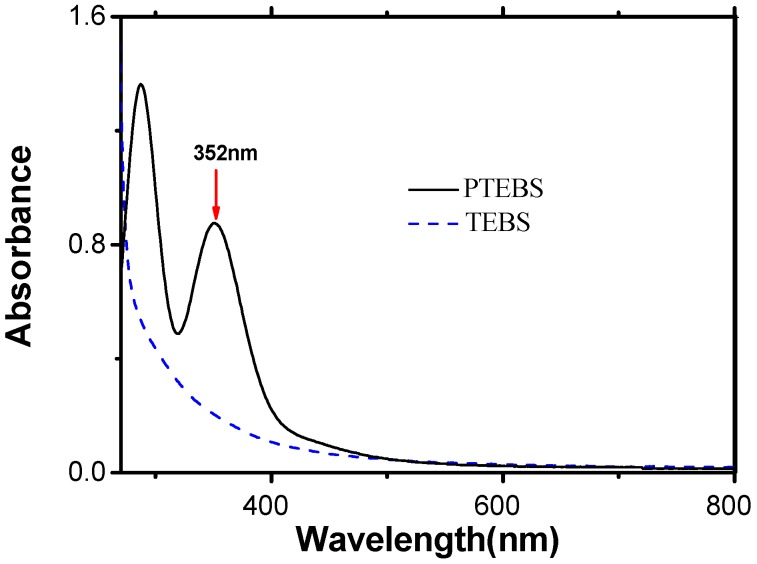
UV–Vis spectra of monomer TEBS and polymer PTEBS.

Moreover, conjugation was also confirmed by the UV–Vis spectrum of the polymer. The UV–Vis spectrums of monomer TEBS and as-synthesized polymer PTEBS are shown in Figure 3. As seen in the figure, the monomer TEBS did not show significant absorption above 300 nm. However, the UV–Vis spectrums of the obtained PTEBS give a peak at 352 nm in the water solution corresponding to the π−π* transition. The spectra of the obtained PTEBS are red-shifted compared to the monomer, attributed to a distribution of conjugation lengths, which further confirms the success of enzyme-catalyzed polymerization. However, the absorption of the PTEBS was limited by finite confinement of the π−conjugated length, caused by limited long-range order, in agreement with the low molecular weight of the PTEBS previously obtained [25].

### 3.2. Thermal Analysis

**Figure 4:**
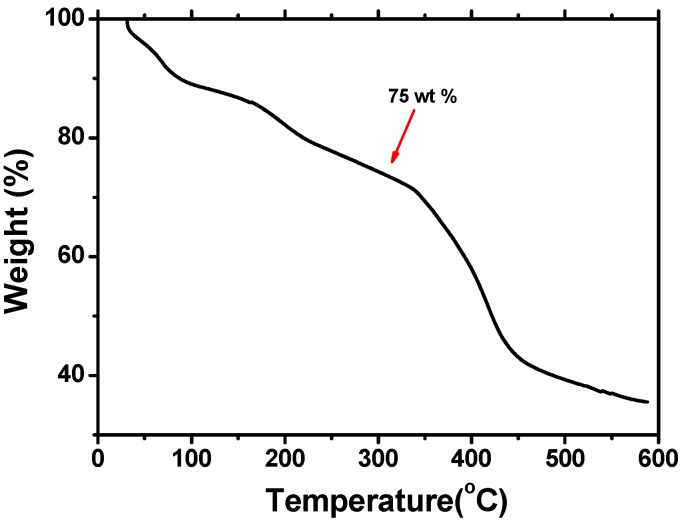
TG curve of polymer PTEBS heated from 0 to 600 °C.

The process of thermal annealing has been demonstrated to considerably increase the performance of the organic solar cells (OSCs) [26,27,28,29]. Therefore, thermal properties of the PTEBS obtained were evaluated by Thermo gravimetric (TG) analyses. The TG trace of the PTEBS solid is shown in the insert of Figure 4. The measurements were performed under nitrogen. The initial decrease in weight percent is due to evaporation of bound water. The enzymatically synthesized PTEBS is fairly stable up to 300 °C (75 wt % is remained) and then starts to decompose. These data show the high thermal stability of the present PTEBS, which is stable over a fairly high range of temperatures. The high thermal stability may be greatly helpful for its potential applications.

### 3.3. The Potential for Using in Solar Cells

The obtained PTEBS has a good solubility in water and ethanol. The benefits of using water as the solvent are numerous, such as easily controlled evaporation rates, environmental friendliness, and improved stability under atmospheric conditions. Water-soluble polythiophene is also compatible with a number of different processing techniques, which help to build high efficiency solar cells using low cost materials and processing methods.

**Figure 5:**
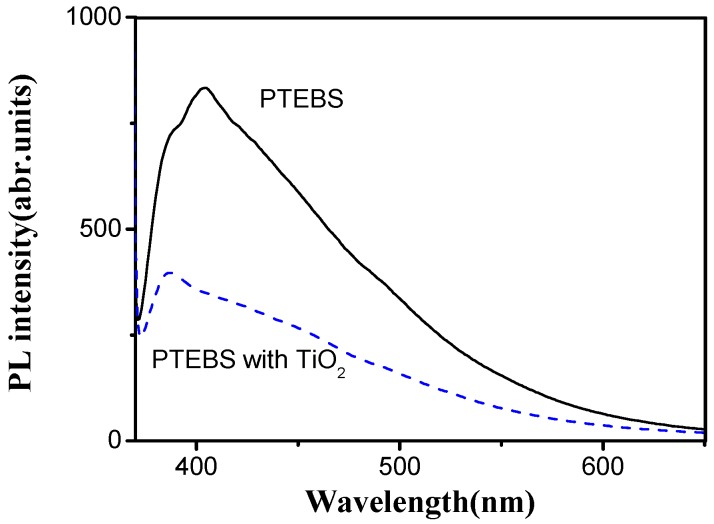
Photoluminescence of PTEBS/TiO_2_ water solution.

Inorganic nano-particles such as titanium dioxide (TiO_2_) often serve as electron acceptors to build hybrid solar cells. Thus, the photovoltaic response of the PTEBS was tested by performing the photoluminescence (PL) intensity of the PTEBS/TiO_2_ composite, which can indicate, and help us understand, the charge separation and transfer in the composites [12]. Figure 5 shows the PL of the pure PTEBS and the PTEBS/TiO_2_ composite that contains PTEBS and TiO_2_ with a ratio of 1:1 by weight. As shown in Figure 5, there is a significant quenching of the fluorescence in the composite as compared to the pure PTEBS, which implies that the excitons dissociate and separate successfully at the interface of PTEBS (donor) and TiO_2_ (acceptor). It renders this obtained PTEBS a very promising candidate of donor material for organic solar cells.

Traditionally, PTEBS was synthesized by chemical oxidation methods. In this work, PTEBS was first synthesized using enzyme-catalyzed method. This enzyme-catalyzed polymerization is a “green synthesis process,” the benefits of which include a simple setting, high yields, and an environmentally friendly route. The obtained PTEBS can build hybrid solar cells with inorganic nano-particles such as TiO_2_ using water as a solvent, which helps towards easily controlled evaporation rates and an environmentally friendly route. The performance of the solar cells can be increased by thermal annealing for the high thermal stability of PTEBS.

## 4. Conclusions

In summary, we have suggested a facile enzyme-catalyzed polymerization method to synthesize a water-soluble poly[2-(3-thienyl)-ethoxy-4-butylsulfonate] (PTEBS), using (3-thienyl)-ethoxy-4-butylsulfonate (TEBS) as a monomer, a horseradish peroxidase (HRP) enzyme as a catalyst, and hydrogen peroxide (H_2_O_2_) as an oxidant. This enzymatic polymerization is a new approach for the synthesis of PTEBS, the benefits of which include a simple setting, high yields, and an environmentally friendly route. These characteristics are indicative of the “green synthesis process” nature of the enzymatic catalysis for developing new polymeric materials. The present PTEBS has a good solubility in water and ethanol, and the excitons dissociate and separate successfully at the interface of PTEBS and TiO_2_, which help to build solar cells using green processing methods.

## Figures and Tables

**Table 1:** Enzyme-catalyzed polymerization of TEBS at different reaction conditions ^1^.

Entry	Reaction temperature (°C)	Reaction time (h)	Amount of HRP (mg)	Molar ratio of TEBS:H_2_O_2_	Yield (%)
1	5	16	0.45	1:1	53
2	25	16	0.45	1:1	40
3	5	16	2.25	1:1	56
4	5	24	0.45	1:1	54
5	5	16	0.45	2:1	21
6	5	16	0.45	1:2	38

^1^ The amount of monomer TEBS is 11 mmol.

## Supplementary Materials

The following are available online at www.mdpi.com/2073-4360/8/4/139/s1: Figure S1: ^1^H NMR spectrum of (3-thienyl)-ethoxy-4-butylsulfonate (TEBS); Figure S2: ^1^H NMR spectrum of poly[2-(3-thienyl)-ethoxy-4-butylsulfonate] (PTEBS); Figure S3: GPC trace of PTEBS.

## References

[1] Chen S.A., Ni J.M. (1992). Structure/properties of conjugated conductive polymers. 1. Neutral poly(3-alkyl thiophene). Macromolecules.

[2] Yamamoto T., Komarudin D., Arai M., Lee B.L., Suganuma H., Asakawa N., Inoue Y., Kubota K., Sasaki S., Fukuda T. (1998). Extensive studies on π-stacking of poly(3-alkylthiophene-2,5-diyl)s and poly(4-alkylthiazole-2,5-diyl)s by optical spectroscopy, NMR analysis, light scattering analysis, and X-ray crystallography. J. Am. Chem. Soc..

[3] Kim Y., Cook S., Tuladhar S.M., Choulis S.A., Nelson J., Durrant J.R., Bradley D.D.C., Giles M., Mcculloch I., Ha C.S. (2006). A strong regioregularity effect in self-organizing conjugated polymer films and high-efficiency polythiophene: Fullerene solar cells. Nat. Mater..

[4] Karahka M., Kreuzer H.J. (2011). Conduction and electrostriction of polymers induced by high electric fields. Polymers.

[5] Zhao Y., Wang X., Shu F. (2011). Investigation of multi-donor bulk-heterojunction photovoltaic cells based on P3HT:PCBM system. Sol. Energ. Mat. Sol. Cells.

[6] Chen T.A., Wu X., Rieke R.D. (1995). Regiocontrolled synthesis of poly(3-alkylthiophenes) mediated by rieke zinc: Their characterization and solid-state properties. J. Am. Chem. Soc..

[7] Pal S., Nandi A.K. (2005). Cocrystallization mechanism of poly(3-alkyl thiophenes) with different alkyl chain length. Polymer.

[8] Chen L., Kim B., Nishino M., Gong J.P., Osada Y. (2000). Environmental responses of polythiophene hydrogels. Macromolecules.

[9] Mwaura J.K., Pinto M.R., Witker D., Ananthakrishnan N., Schanze K.S., Reynolds J.R. (2005). Photovoltaic cells based on sequentially adsorbed multilayers of conjugated poly(*p*-phenylene ethynylene)s and a water-soluble fullerene derivative. Langmuir.

[10] Wang H., Xia B., Yan Y., Li N., Wang J.Y., Wang X. (2013). Water-soluble polymer exfoliated graphene: As catalyst support and sensor. J. Phys. Chem. B.

[11] Qiao Q., McLeskey J.T. (2005). Water-soluble polythiophene/nanocrystalline TiO_2_ solar cells. Appl. Phys. Lett..

[12] McLeskey J.T., Qiao Q. (2006). Hybrid solar cells from water-soluble polymers. Int. J. Photoenergy.

[13] Tran-Van F., Carrier M., Chevrot C. (2004). Sulfonated polythiophene and poly(3,4-ethylenedioxythiophene) derivatives with cations exchange properties. Synth. Met..

[14] Cabarcos E.L., Carter S.A. (2005). Effect of the molecular weight and the ionic strength on the photoluminescence quenching of water-soluble conjugated polymer sodium poly[2-(3-thienyl)ethyloxy-4-butylsulfonate]. Macromolecules.

[15] Gross R.A., Kumar A., Kalra B. (2001). Polymer synthesis by *in vitro* enzyme catalysis. Chem. Rev..

[16] Bruno F.F., Fossey S.A., Nagarajan S., Nagarajan R., Kumar J., Samuelson L.A. (2006). Biomimetic synthesis of water-soluble conducting copolymers/homopolymers of pyrrole and 3,4-ethylenedioxythiophene. Biomacromolecules.

[17] Kadokawa1 J., Kobayashi S. (2010). Polymer synthesis by enzymatic catalysis. Curr. Opin. Chem. Biol..

[18] Kazariya A., Matsumura S. (2012). Enzymatic synthesis and crosslinking of novel high molecular weight polyepoxyricinoleate. Polymers.

[19] Mohan T., Rathner R., Reishofer D., Koller M., Elschner T., Spirk S., Heinze T., Stana-Kleinschek K., Kargl R. (2015). Designing hydrophobically modified polysaccharide derivatives for highly efficient enzyme immobilization. Biomacromolecules.

[20] Kobayashi S., Uyama H., Takamoto T. (2000). Lipase-catalyzed degradation of polyesters in organic solvents. A new methodology of polymer recycling using enzyme as catalyst. Biomacromolecules.

[21] Kobayashi S., Makino A. (2009). Enzymatic polymer synthesis: An opportunity for green polymer chemistry. Chem. Rev..

[22] Sandy M., Carter-Franklin J.N., Martin J.D., Butler A. (2011). Vanadium bromoperoxidase from Delisea pulchra: Enzyme-catalyzed formation of bromofuranone and attendant disruption of quorum sensing. Chem. Commun..

[23] Duan L., Zhao Y., Guo F., Liu W., Hou C., Ni Z. (2014). Enzymatic-catalyzed polymerization of water-soluble electrically conductive polymer PEDOT:PSS. Polym. Adv. Technol..

[24] Hu X., Xu L. (2000). Structure and properties of 3-alkoxy substituted polythiophene synthesized at low temperature. Polymer.

[25] Hiorns R.C., de Bettignies R., Leroy J., Bailly S., Firon M., Sentein C., Khoukh A., Preud’homme H., Dagron-Lartigau C. (2006). High molecular weights, polydispersities, and annealing temperatures in the optimization of bulk-heterojunction photovoltaic cells based on poly(3-hexylthiophene) or poly(3-butylthiophene). Adv. Funct. Mater..

[26] Ma W., Yang C., Gong X., Lee K., Heeger A.J. (2005). Thermally stable, efficient polymer solar cell with nanoscale control of the interpenetrating network morphology. Adv. Funct. Mater..

[27] Yang X., Loos J., Veenstra S.C., Verhees W.J.H., Wienk M.M., Kroon J.M., Michels M.A.J., Janssen R.A.J. (2005). Nanoscale morphology of high-performance polymer solar cells. Nano. Lett..

[28] Michinobu T., Fujita H. (2010). Postfunctionalization of alkyne-linked conjugated carbazole polymer by thermal addition reaction of tetracyanoethylene. Materials.

[29] Zhao Y., Duan L., Liu J., Xu Q., Ni Z. (2013). Optimisation of thermal annealing parameters for different thickness of active layers based on polymer/fullerene bulk heterojunction solar cells. Mater. Res. Innov..

