# Supplementary material for: Enzyme-Catalyzed Synthesis of Water-Soluble Conjugated Poly[2-(3-thienyl)-Ethoxy-4-Butylsulfonate]
Source: Polymers (Basel). 2016 Apr 13;8(4):139. doi: 10.3390/polym8040139 (PMC6431961; doi:10.3390/polym8040139)
Supplement: Supplementary file 1 [file polymers-08-00139-s001.pdf]

# Supplementary Materials: Enzyme-Catalyzed Synthesis of Water-Soluble Conjugated Poly[2-(3-thienyl)-Ethoxy-4-Butylsulfonate]

**Yun Zhao, Hongyan Zhu, Xinyang Wang, Yingying Liu, Xiang Wu, Heyuan Zhou and Zhonghai Ni**

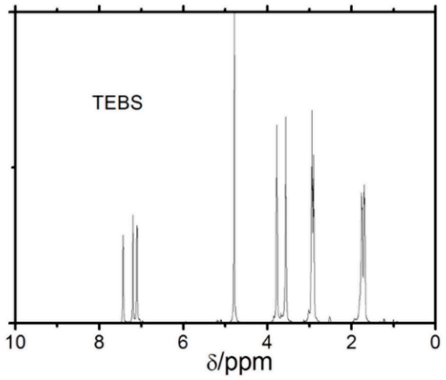


**Figure S1.** $^1$H NMR spectrum of (3-thienyl)-ethoxy-4-butylsulfonate (TEBS).

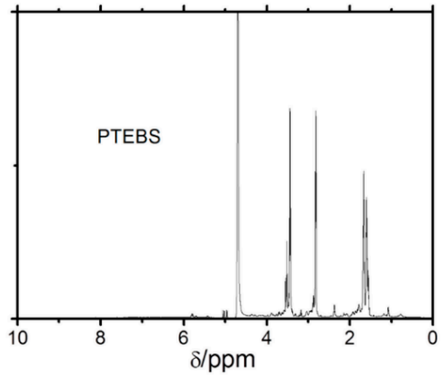


**Figure S2.** $^1$H NMR spectrum of poly[2-(3-thienyl)-ethoxy-4-butylsulfonate] (PTEBS).

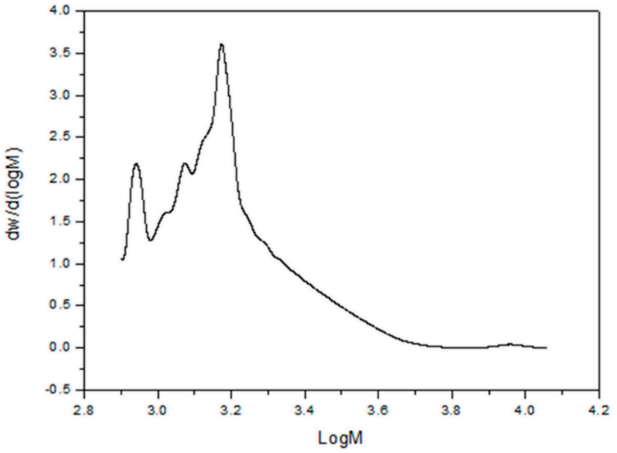


**Figure S3.** GPC trace of PTEBS.

© 2016 by the authors; licensee MDPI, Basel, Switzerland. This article is an open access article distributed under the terms and conditions of the Creative Commons by Attribution (CC-BY) license (http://creativecommons.org/licenses/by/4.0/).
